# 2′,6′-Dimethylphenylalanine: A Useful Aromatic Amino Acid Surrogate for Tyr or Phe Residue in Opioid Peptides

**DOI:** 10.1155/2012/498901

**Published:** 2012-04-04

**Authors:** Yusuke Sasaki, Akihiro Ambo

**Affiliations:** Department of Biochemistry, Tohoku Pharmaceutical University, 4-4-1 Komatsushima, Aoba-ku, Sendai 981-8558, Japan

## Abstract

Two aromatic amino acids, Tyr^1^ and Phe^3^ or Phe^4^, are important structural elements in opioid peptides because they interact with opioid receptors. The usefulness of an artificial amino acid residue 2′,6′-dimethylphenylalanine (Dmp) was investigated as an aromatic amino acid surrogate for several opioid peptides, including enkephalin, dermorphin, deltorphin, endomorphin, dynorphin A, and nociceptin peptides. In most peptides, substitutions of Phe^3^ by a Dmp residue produced analogs with improved receptor-binding affinity and selectivity, while the same substitution of Phe^4^ induced markedly reduced receptor affinity and selectivity. Interestingly, replacement of Tyr^1^ by Dmp produced analogs with unexpectedly high affinity or produced only a slight drop in receptor affinity and bioactivity for most peptides. Thus, Dmp is also a useful surrogate for the N-terminal Tyr residue in opioid peptides despite the lack of a phenolic hydroxyl group, which is considered necessary for opioid activity. The Dmp^1^-substituted analogs are superior to 2′,6′-dimethyltyrosine (Dmt)^1^-substituted analogs for high receptor selectivity since the latter generally have poor receptor selectivity. Thus, Dmp is very useful as an aromatic amino acid surrogate in opioid peptides and may be useful for developing other novel peptide mimetics with high receptor specificity.

## 1. Introduction

Three major types of opioid receptors, *μ*, *δ*, and *κ*, have been cloned and assigned to the superfamily of rhodopsin-like G-protein-coupled receptors [1–3]. The *μ*-receptors are involved in supraspinal analgesia, respiratory depression, euphoria, sedation, decreased gastrointestinal motility, and physical dependence [4]. The *δ*-receptors appear to affect cardiovascular function, contribute to analgesia, and cause changes in affective behavior [4]. The *κ*-receptors are responsible for spinal analgesia, miosis, a modest degree of sedation, and some respiratory depression [4]. *In vivo,* opioid peptides exert pharmacological actions *via* the opioid receptors. Enkephalins (Tyr-Gly-Gly-Phe-Leu/Met) [5] and endomorphins (Tyr-Pro-Phe/Trp-Phe-NH_2_) [6] are endogenous ligands for the *δ*- and *μ*-opioid receptors, respectively. Dynorphin A (DYN: Tyr-Gly-Gly-Phe-Leu-Arg-Arg-Ile-Arg-Pro-Lys-Leu-Lys-Trp-Asp-Asn-Gln) is the endogenous ligand of the *κ*-opioid receptor [7]. Dermorphin (DM, Tyr-D-Ala-Phe-Gly-Tyr-Pro-Ser-NH_2_) [8] and [D-Ala^2^]deltorphin II (DT, Tyr-D-Ala-Phe-Glu-Val-Val-Gly-NH_2_) [9] are naturally occurring opioid peptides isolated from South American frogs and possess high selectivity toward *μ*- and *δ*-opioid receptors, respectively.

A fourth cloned member of the opioid-receptor family is the opioid receptor-like 1 (ORL1) receptor that shares high sequence homology with traditional opioid receptors [10]. The heptadecapeptide nociceptin (NOC) [11] or orphanin FQ [12] (NOC: Phe-Gly-Gly-Phe-Thr-Gly-Ala-Arg-Lys-Ser-Ala-Arg-Lys-Leu-Ala-Asn-Gln) was discovered as an endogenous ligand for the ORL1 receptor. NOC and DYN have a similar structural feature including the N-terminal tetrapeptide sequence Phe-Gly-Gly-Phe in NOC and Tyr-Gly-Gly-Phe in DYN and the existence of basic residues, although with different distributions, at the C-terminal. Despite the sequence homology, NOC and DYN have different pharmacological profiles [13, 14]. NOC possesses hyperalgesia and allodynia activity when applied supraspinally at low doses, while spinal delivery of NOC causes analgesia at high doses [11, 13–15]. Two aromatic amino acids, Phe^1,4^ at the N-terminal of NOC [14, 16, 17] and Tyr^1^ and Phe^4^ in DYN [18, 19], are important residues required for receptor binding and/or biological activity and are needed for discriminating between them. In particular, the presence of N-terminal Phe^1^ is indispensable for NOC activity, and the C-terminal half of NOC may serve as a domain that prevents binding to opioid receptors [20].

**Figure 1:**
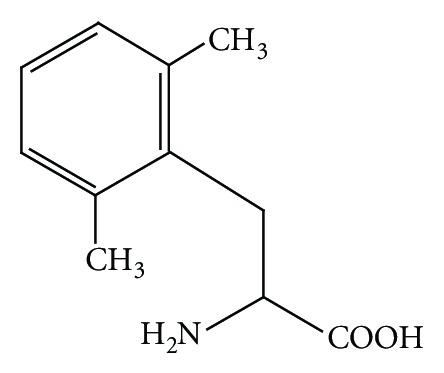
Structure of 2′,6′-dimethylphenylalanine (Dmp).

In the field of opioid peptides, a number of synthetic analogs have been prepared based on structure-activity studies focusing on the aromatic amino acids Tyr^1^ and either Phe^3^ or Phe^4^, which are important structural elements that interact with opioid receptors [21]. Among these, the most active analogs were those that substituted 2′,6′-dimethyltyrosine (Dmt) for Tyr^1^, which vastly improved opioid receptor binding affinity [22–36]. Structure-activity relation studies of opioid peptides using Dmt revealed that introduction of Dmt^1^ could improve receptor affinity and opioid potency. In addition, combination of Dmt with 1,2,3,4-tetraisoquinoline-3-carboxylic acid (Tic), Dmt-Tic pharmacophore, also produced potent *δ*-selective antagonists, including Dmt-Tic-OH [31, 32], N,N′-diMeDmt-Tic-OH [33], DIPP-NH_2_ [34], and DIPP [Ψ] [35]. The 2,6-dimethylation of the aromatic moiety in Leu-enkephalin (ENK) imparted high enzymatic stability to the peptide [36]. These findings prompted a study to modify a Phe aromatic moiety at position 3 or 4 of opioid peptides through 2,6-dimethylation because no derivatives with phenyl ring-methylated Phe incorporated into opioid peptides have been reported, only other biologically active peptides have been prepared [37, 38]. The usefulness of incorporating the artificial aromatic amino acid, 2′,6′-dimethylphenylalanine (Dmp) (Figure 1) as an aromatic amino acid surrogate in opioid peptides to develop opioid ligands specific for opioid receptors was investigated. This paper includes all studies that involved substitution of the Dmp residue into opioid peptides reported in the last decade.

## 2. Preparation of 2′,6′-Dimethylphenylalanine (Dmp)

**Scheme 1:**
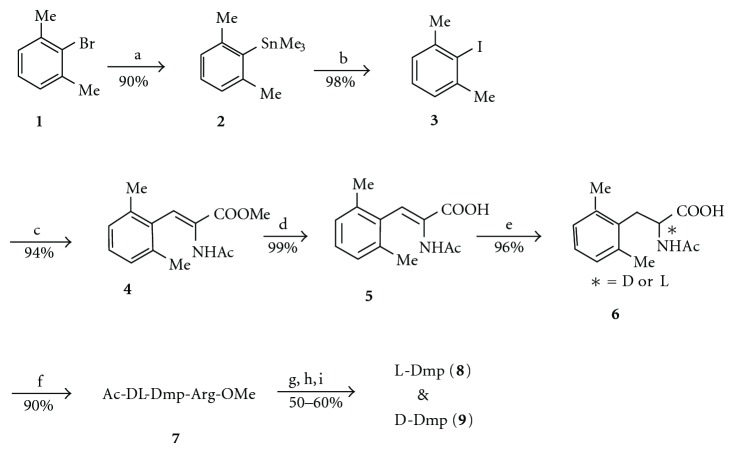
Synthetic route to L-Dmp and D-Dmp, (a) Me_3_SnNa, DME, ice-salt bath, 2 h; (b) I_2_/THF, rt, 3 h; (c) methyl 2-acetamidoacrylate/Pd(OAc)_2_/Et_3_N/MeCN, (2-MeC_6_H_4_)_3_P, reflux, 24 h; (d) 1 M NaOH/dioxane, rt, 2 h; (e) H_2_ (4 kgf/cm^2^)/10% Pd-C/AcOH, 70°C, 48 h; (f) HCl*·*Arg-OMe/Et_3_N/DCC/HOBt/DMF, 0°C to rt, 5 h; (g) preparative HPLC; (h) concd HCl, reflux, 8 h; (i) pH 4–6/H_2_O.

Initially, Dmp and D-Dmp were synthesized by the route illustrated in Scheme 1 [39]. Commercially available **1** reacted with sodium trimethyl stannane according to the method of Yamamoto et al. [40], followed by reaction with iodine according to the method of Ohno et al. [41]. A key intermediate, 2-iodo-*m*-xylene (**3**), reacted with methyl 2-acetamidoacrylate by Dygos' method [42], which led to **4**, followed by saponification to yield **5**. Catalytic hydrogenation of **5** yielded racemic Ac-Dmp (**6**). For optical resolution, **6** was converted to its dipeptide derivatives, Ac-DL-Dmp-Arg-OMe (**7**), which were easily separated into diastereoisomers by preparative HPLC. Acid hydrolysis and neutralization of each isomer yielded Dmp (**8**) and D-Dmp (**9**). The L and D configurations were determined using L-amino acid oxydase according to a method reported by Toth et al. [43].

In addition, Li et al. prepared Dmp using the asymmetric synthetic method of Dygos et al. [42], which was applied to the endomorphin analogs [44].

## 3. Dmp Replacement of Phenylalanine Residue at Position 3 or 4 in Opioid Peptides

The usefulness of Dmp was first investigated as a surrogate for Phe^4^ in ENK analogs [39]. The receptor-binding affinities of synthetic analogs were determined using rat brain, as previously reported [45]. As shown in Table 1, replacement of Phe^4^ in ENK by Dmp led to analog **10 **with *μ*-receptor affinity comparable to ENK, but with approximately 12-fold reduction in *δ*-receptor affinity, resulting in a change of preferential receptor from *δ* to *μ*. The D-Dmp replacement of ENK (**11**) induced significant reduction in binding affinity for both receptors. Combined replacement of Dmp^4^ and Dmt^1^ produced **12** with markedly improved affinities for both receptors, 40- and 110-fold greater in affinity than **10 **for the *μ*- and *δ*-receptors, respectively. However, **12 **possessed 5-fold lower affinity than that of [Dmt^1^]ENK for both receptors, possibly due to slight changes in the active conformation by simultaneous dimethylation of two aromatic moieties. D-Dmp^4^ replacement of **12** led to **13**, which retained *μ* affinity equivalent to that of ENK and modest *δ* affinity, demonstrating the effectiveness of Dmt^1^ replacement for maintaining high receptor affinity. *In vitro* biological activity of ENK analogs was evaluated using isolated guinea pig ileum (GPI) and mouse vas deferens (MVD) tissue samples, as previously reported [46]. The GPI tissue contains predominantly *μ*-receptors, while MVD tissue contains *δ*-receptors [47]. As shown in Table 1, compound **10** possessed 8- and 30-fold lower activity compared to ENK in the GPI and MVD assays, respectively. In contrast, **11** was devoid of activity in both assays, as expected from the binding data. Analog **13** also lacked activity in both assays, even though this analog showed potent *μ* affinity and modest *δ* affinity. This analog turned out to be a potent *μ*-antagonist and a weak *δ*-antagonist. The pA_2_ values of **13** were 6.90 against EM2 as a *μ* agonist in the GPI assay and 5.57 against DT as a *δ* agonist, in the MVD assay. The results of **13** are in line with observations that Dmt-D-Phe-NH_2_ and its C-terminally extended analog are *μ*-receptor antagonists [48].

 Effects of Dmp substitution for phenylalanine at position 3 or position 4 in EM2 were examined (Table 1) [49]. The EM2 possessed great affinity and selectivity for the *μ*-receptor. Interestingly, Dmp substitution for Phe^3^ in EM2 (**14**) produced a compound with 10-fold greater affinity than that of EM2 for both the *μ*- and *δ*-receptors and still retained high *μ*-receptor selectivity comparable to that of EM2. The Dmp substitution of Phe in position 4 (**16**), however, resulted in a 23-fold decrease in *μ* affinity and a slight increase in *δ* affinity, resulting in a significant decrease in *μ*-receptor selectivity. The introduction of D-Dmp at either position 3 or 4 (**15** or **17**, resp.) resulted in a significant decrease in *μ* affinity and selectivity, which agreed with the results from D-Phe-replaced analogs [50]. Analog **15** retained moderate *μ* affinity with a *K*i value of 2.4 nM, whereas **16** and **17** exhibited significantly decreased *μ* affinity. In the* in vitro *assay, **14** exhibited considerably greater GPI potency than EM2 as expected; however, this analog exhibited more potent MVD activity than that expected from *δ*-binding. This may be due to *μ*-receptors, which coexist in the MVD tissues, because the high MVD potency was strongly inhibited by the specific *μ*-receptor antagonist CTAP [51]. A similar trend was observed with other *μ*-receptor ligands [52, 53]. These results suggest that Dmp substitution of Phe^3^ of EM2 promotes *μ*-receptor specificity and that Phe^3^ is more amenable to Dmp or its D-isomer substitution compared to Phe^4^.

 Use of Dmp as a Phe surrogate in DM and DT heptapeptides was also examined [52]. Replacement of Phe by Dmp in the *μ*-specific ligand DM (**18**) induced a significant increase (170-fold) in *μ* affinity and only a modest increase in *δ* affinity, resulting in marked improvement of *μ*-receptor selectivity. The D-Dmp^3^ replacement (**19**), however, resulted in marked decrease in both *μ* and *δ* affinities. Interestingly, the Dmp^3^ replacement in *δ*-specific ligand DT produced **20** with a 22-fold increase in *δ* affinity and a 3-fold decrease in *μ* affinity, resulting in a 75-fold increase in *δ*-receptor selectivity with unprecedented *δ*-receptor selectivity (*μ*/*δ* = 1,045,714). The configurational inversion of Dmp in DT (**21**) was detrimental to *δ*-receptor selectivity. Results of the* in vitro* bioassay of these analogs showed that **18** exhibited a slight increase in GPI potency and a greater increase in MVD potency, while **19 **showed marked decreases in both assays as expected from binding affinities. The discrepancy between the degree of increase (3-fold) in the GPI assay observed with **18** and the *μ*-binding data (170-fold) may be due to differences in *μ*-receptors in the brain and peripheral tissues. The Dmp^3^-substituted DT analog **20 **showed markedly increased MVD potency, resulting in a very high GPI/MVD ratio of 304,772. As expected, D-Dmp^3^-substituted DT (**21**) possessed very low MVD potency. Analogs **18** and **20** are among the most potent and selective ligands for *μ*- and *δ*-opioid receptors, respectively, and therefore are candidates for investigations of opioid systems.

 A dermorphin tetrapeptide analog, Tyr-D-Arg-Phe-*β*Ala-NH_2_ (YRFB), is a highly potent and selective ligand for the *μ*-opioid receptor [53]. The usefulness of Dmp replacement for Phe^3^ in this tetrapeptide was examined [54]. Substituting Dmp for Phe^3^ in YRFB (**22**) induced a 5-fold increase in *μ*-receptor affinity without significant change in *δ*-receptor affinity, as compared to the parent peptide. Results from the GPI assay using this compound coincided well with the binding data, but a slight increase in activity in the MVD assay was found. As shown in Table 2, low *K*_e_ values for the *μ*-antagonist CTAP and high *K*_e_ values for the *δ* antagonist N, N(Me)_2_Dmt-Tic-OH [33], against **22** suggest that the GPI activity of these analogs occurred mainly via the *μ*-opioid receptor. Analog **22** was also tested for analgesic activity in the formalin test in mice and was compared to results for YRFB and morphine. As shown in Table 3, subcutaneous injection of this analog produced dose-dependent antinociceptive activity in mice in both the first and second phases. Its analgesic activity was approximately 40- and 70-fold more potent than that of morphine in the first and second phases, respectively. These results indicate that Dmp is effective as a Phe surrogate for improving functional activity and maintaining *μ*-selectivity [52]. In contrast, [D-Dmp^3^] YRFB (**23**) exhibited *μ*-receptor affinity similar to the parent peptide, but it exhibited an order of magnitude lower GPI potency.

 Next, the effect of Dmp replacement of N-terminal aromatic residues in DYN and NOC was compared [55]. Six analogs (**26–31**) containing Dmp in position 1 and/or 4 of DYN (1-13)-NH_2_ and NOC (1-13)-NH_2_ were synthesized and tested for their binding affinity to opioid receptors derived from rat (*μ*- and *δ*-receptors) or guinea pig (*κ*-receptor) brains and to membrane preparations derived from HEK293 cells expressing human ORL-1 receptor. Results are shown in Table 4. In a series of DYN(1-13)-NH_2_ analogs, the parent peptide DYN(1-13)-NH_2_ showed high affinity toward *κ*-, *μ*-, and *δ*-opioid receptors with *κ*-receptor selectivity, that is, an IC_50_ ratio of 1/15.6/40.1 and significantly low affinity toward the ORL1 receptor, similar to an observation of intact DYN [11, 20]. Dmp^4^ replacement afforded **26**, which had greater *κ*-opioid receptor affinity than that of the parent peptide and significantly improved *κ*-receptor selectivity (IC_50_ ratios: 1(*κ*)/509(*μ*)/21159(*δ*) versus DYN(1-13)-NH_2_, 1(*κ*)/15.6(*μ*)/40.1(*δ*)). Compound **26** exhibited an order of magnitude decrease in affinity, indicating that the Dmp^4^ modification in DYN peptides is detrimental to ORL1-receptor affinity, as was observed with NOC peptides. NOC (1-13)-NH_2_ possessed high ORL1 receptor affinity and poor affinity for *κ*-, *μ*-, and *δ*-opioid receptors. The Dmp^4^-NOC analog (**29**) showed a 70-fold decrease in ORL1 affinity without significant changes in affinity toward the opioid receptors. These results indicate the critical importance of the Phe^4^ residue for interactions with the ORL1 receptor. A Dmp residue at this position appears to influence the conformation of the NOC peptide by 2′,6′-dimethylation of the Dmp side chain aromatic moiety. This occurs because, according to the proposed model of the ORL1 receptor and its complex with NOC, the Phe^4^ residue of NOC located at a hydrophobic pocket in a cavity formed by TM helices 3, 5, 6, and 7 and the Phe^4^ side chain interact with Phe^220^ of the ORL1 receptor through an edge-face interaction [56]. Two methyl groups on Dmp^4^ may interfere with the receptor interaction due to a reduction in conformational flexibility and/or enhanced lipophilicity. To further examine the usefulness of Dmp-containing DYN peptides as *κ*-opioid receptor ligands, the *in vitro* bioactivity of DYN peptides was determined using the GPI assay (Table 5). Contrary to the high *κ*-opioid receptor-binding profile, **26** exhibited unexpectedly low GPI potency, which was one order of magnitude lower than the parent peptides. Low *K*_e_ values for the *κ*-receptor antagonist and high *K*_e_ values for the *μ*- and *δ*-antagonists of **26** suggest that the GPI activity of these analogs occurred mainly via the *κ*-opioid receptor.

## 4. Dmp Replacement of N-Terminal Tyr Residue in Opioid Peptides

 The usefulness of Dmp^1^ substitution for Tyr^1^ in the *δ*-opioid receptor-selective ligands, ENK and DT, and the *μ*-opioid receptor-selective ligands, EM2 and YRFB, has been investigated [49, 53, 57]. Results of receptor-binding and *in vitro* assays are shown in Table 6. The replacement of Tyr^1^ by Dmp in ENK led to **32**, which possessed similar receptor affinity and selectivity as ENK, whereas Dmt^1^ replacement produced marked increases in both *μ* and *δ* affinities but did not increase receptor selectivity. Replacement of Phe^1^ in ENK (**33**) decreased the affinity by 50- and 70-fold at the *δ*- and *μ*-receptors, respectively. Replacement of Tyr^1^ by Dmp in DT (**34**) markedly decreased the binding affinity and selectivity toward the *δ*-receptor. Introduction of the inverse configuration at this position (**35**) markedly reduced *δ* affinity and selectivity. In contrast, [Dmt^1^]DT (**36**) possessed a 50-fold increase in *δ* affinity and a 1200-fold increase in *μ* affinity, resulting in substantial reduction in *δ*-receptor selectivity, which agrees with previous results [28]. [Phe^1^]DT (**37**) retained significant *δ* affinity and good *δ* selectivity, similar to the results for [Phe^1^] deltorphin I [58]. Analog **32** possessed lower MVD and GPI potency compared to ENK but showed significant MVD potency and GPI/MVD selectivity in agreement with the binding data. Analog **34 **exhibited 2-fold greater potency for MVD and a greater GPI/MVD ratio compared to DT (selectivity ratio: 14,835 versus 9342) or [Dmt^1^]DT (**36**) (selectivity ratio: 14,835 versus 1700). The [Phe^1^]DT (**37**) exhibited a 20-fold decrease in MVD potency compared to DT but retained significant potency and MVD specificity. These results demonstrate that the Dmp^1^ peptide is superior to the corresponding Dmt^1^ peptide in receptor selectivity because the latter generally possesses poor receptor selectivity.

 In EM2 analogs, replacement of Tyr^1^ by Dmp led to **38**, which showed a 4-fold reduction, retaining significant *μ* affinity. Note that **38** retained high potency for *μ*-receptors despite the lack of a phenolic hydroxyl group at the N-terminal, which agreed with results for YRFB analogs [53]. The D-Dmp^1^- or Phe^1^-substituted analogs of EM2 (**39** and** 40**, resp.) showed a significant decrease in GPI potency, which was expected from the binding affinity. In contrast, **39** and **40 **were more than 100-fold less potent than EM2. Results from analog **38** supported the observation that a Dmp residue can mimic the N-terminal Tyr of opioid peptides [53]. 

Replacing Tyr with Dmp at position 1 in YRFB (**24**) produced greater *μ* affinity and considerably lower *δ* affinity compared to YRFB and improved *μ*-receptor selectivity by 15-fold. This compound, however, showed slightly lower GPI and MVD potency compared to YRFB. The D-Dmp substitution for Tyr^1^ (**41**) markedly reduced affinities for both receptors and for *in vitro* biological activity, suggesting that the L-configuration at this position is crucial for receptor interactions. The dual substitutions of Dmp for the aromatic amino acids at positions 1 and 3 produced** 25 **with binding affinity and selectivity for the *μ*-receptor that were slightly improved relative to those of **22** or **24**. This analog also showed slightly greater GPI potency than YRFB. As shown in Table 2, the low *K*_e_ value found for the *μ*-receptor selective antagonist CTAP in the GPI assay demonstrated inhibition of the high activity of Dmp^1^-containing analogs (**24** and **25**) and suggests that the activity is mediated via *μ*-opioid receptors. The low *K*_e_ values for CTAP in the MVD assay indicate its inhibition of the analogs MVD activity, but the *δ*-receptor selective antagonist *N,N*(Me)_2_Dmt-Tic-OH did not inhibit this activity. This result may be due to *μ*-receptor cooccurring with the *δ*-receptor in MVD tissue. Analogs **24** and **25** retained high *μ*-receptor affinity and potent GPI activity despite the lack of a phenolic hydroxyl group in the side chain of the N-terminal residue, which is considered crucial for binding and activating opioid receptors. However, some cyclic somatostatin- or DPDPE-based analogs possess high affinity for and/or potency toward the *μ*-receptor despite the absence of this group at the N-terminal residue [59–63]. Analogs **24** and **25** are examples of linear peptides lacking an N-terminal phenolic hydroxyl group but possess high opioid activity. The present results support reports of the interactions of cyclic compounds that indicate the Tyr hydroxyl moiety at the N-terminal residue of opioid peptides is not an absolute requirement for interaction with opioid receptors and signal transduction. Because replacing the Tyr^1^ residue with Phe (**42**) drastically reduced *μ*-receptor affinity and GPI potency, effects of Dmp substitution on receptor interactions are attributable mainly to enhanced hydrophobicity and/or increased conformational stability of the side chain of the aromatic ring. The basic functional group of the D-Arg residue at position 2 may also be responsible for the potent receptor interaction because the affinity of **41** and **42** was very low, but significant affinity for the *μ*-receptor was retained. In addition, the Dmt^1^-substituted YRFB exhibited great affinity for both the *μ*- and *δ*-receptors, which often resulted in low receptor selectivity. Such trends have also been observed with other Dmt^1^-substituted opioid peptides [39, 64, 65]. In contrast, substitution of Dmp^1^ for Tyr^1^ improved *μ*-receptor selectivity exclusively, a result distinct from the effects of Dmt^1^substitution. In the formalin test in mice, analog **24** also exhibited approximately 3-fold (first phase) and 5-fold (second phase) greater potency than that of morphine, but the potencies were approximately 3-fold less than those of YRFB. The analgesic potencies of these analogs correlated well with their GPI potencies.

In a series of DYN(1-13)-NH_2_ analogs, Dmp^1^ replacement afforded **27** with greater *κ*-opioid receptor affinity than that of the parent peptide; Dmp^1^ replacement also significantly improved *κ*-receptor selectivity (IC_50_ ratios: **27**, 1(*κ*)/293(*μ*)/180(*δ*) versus DYN(1-13)-NH_2_, 1(*κ*)/15.6(*μ*)/40.1(*δ*)). These results support our recent finding that Dmp is an effective surrogate for the Tyr^1^ residue in opioid peptides [49, 53, 57]. Analog **27**, however, exhibited low GPI potency two orders of magnitude less than DYN(1-13)-NH_2_. Low *K*_e_ values for the *κ*-receptor antagonist nor-BNI suggests that its GPI activity occurred mainly via the *κ*-opioid receptor, similar to the observations for **26**. The discrepancy between *κ*-opioid receptor binding and GPI potency observed with **27** can be attributed to the lack of hydroxyl side chains on the N-terminal residue because the [Dmt^1^]DYN peptide was as active as the parent peptide in a GPI assay [66]. Similar results have been reported for the Phe^1^-DYN (1–11) peptide [67]. Unexpectedly, however, **27** possessed 3-fold greater affinity toward the ORL1 receptor, whereas **26** exhibited an order of magnitude decrease in ORL1 receptor affinity, indicating that Dmp^4^ modification in DYN peptides is detrimental to ORL1-receptor affinity. Simultaneous Dmp replacements in positions 1 and 4 **(28**) resulted in a two order of magnitude decrease in *κ*-receptor affinity and dramatically reduced GPI potency with loss of receptor selectivity. These results indicate that the N-terminal phenolic hydroxyl group of the DYN peptide is not mandatory for *κ*-receptor binding but is critically important for receptor activation.

As shown in Table 4, Dmp^1^-NOC peptide (**30**) possessed high ORL1 receptor affinity comparable to the parent peptide NOC (1-13)-NH_2_. Interestingly, this analog exhibited improved affinity toward the three opioid receptors, with 5- and 16-fold improved affinity for the *κ*- and *μ*-receptors, respectively, perhaps due to the effect of Dmp^1^, which can mimic Tyr^1^ in some opioid peptides without a substantial decrease in receptor affinity [53, 58]. Dmp substitutions in positions 1 and 4 afforded **31** with a moderate decrease in affinity toward the ORL1 and opioid receptors, indicating that a Dmp residue in position 1 can compensate for the decrease caused by the Dmp^4^ substitution.

Substitution of the peptide with an artificial amino acid often improves metabolic stability, which is useful when conducting *in vivo* and *in vitro* studies. As shown in Table 7, Dmp^1^-substituted analogs **27** and **30** showed greater stability toward aminopeptidase M (AP-M) and rat brain synaptosomal enzymes compared to the parent peptides, which suggests the involvement of aminopeptidase(s) in the brain that breaks down these analogs as observed with intact NOC [68, 69] and DYN [70, 71]. However, the stability of the Dmp^4^-substituted analogs (**26** and **29**) was similar to or somewhat less than that of the parent peptide toward rat brain enzymes. These results imply that a Dmp residue in position 4 offers no additional metabolic stability for either peptide and that endopeptidases play a major role in brain metabolism. A doubly Dmp-replaced NOC analog **31** also possessed no additional stability, whereas its counterpart DYN analog **28** possessed improved metabolic stability. Comparison of the metabolism of Dmp-containing NOC and DYN analogs suggested that the NOC peptides generally are more susceptible to aminopeptidases and endopeptidases although other results in human blood have been reported [71].

## 5. Conclusions

The usefulness of the artificial amino acid residue Dmp was investigated as an aromatic amino acid surrogate for opioid peptides and related peptides, including ENK, DM, YRFB, DT, EM2, DYN, and NOC peptides. In most opioid peptides, substitution of Phe^3^ by Dmp produced analogs with improved receptor-binding affinity and selectivity, for example, [Dmp^3^]EM2 (**14**), [Dmp^3^]DM (**18**), [Dmp^3^]DT (**20**), and [Dmp^3^]YRFB (**22**), while substitution by the D-enantiomer resulted in decreased receptor affinity and selectivity. A small analog [D-Dmp^3^]YRFB (**23**) was the only exception because it possessed high *μ* affinity similar to the parent peptide. However, Dmp-substitution in position 4 produced analogs with markedly reduced receptor affinity and selectivity, for example, [Dmp^4^]ENK (**10**), [Dmp^4^]EM2 (**16**), and [Dmp^4^]NOC (1-13)-NH_2_ (**29**), while their D-Dmp^4^-analogs were almost devoid of receptor affinity and opioid activity. [Dmp^4^]DYN(1-13)-NH_2_ (**26**) exceptionally possessed significantly improved receptor affinity for the *κ*-opioid receptor and outstanding *κ*-receptor selectivity. Interestingly, replacement of Tyr^1^by Dmp residue produced analogs with equipotent or only slightly reduced receptor affinity and* in vitro* bioactivity, for example, [Dmp^1^]EM2 (**38**), [Dmp^1^]DT (**34**), [Dmp^1^]YRFB (**24**), and [Dmp^1^]DYN(1-13)-NH_2_ (**27**). Thus, Dmp is also a useful surrogate for the N-terminal Tyr residue in opioid peptides despite the lack of a phenolic hydroxyl group, which has been considered to be indispensable for opioid activity. The Dmp^1^-substituted analogs are superior to Dmt^1^-substituted analogs in opioid receptor selectivity because the Dmt^1^ analogs generally possess outstandingly high affinity to opioid receptors but poor receptor selectivity. These results demonstrate that Dmp is very useful as an aromatic amino acid surrogate in opioid peptides and may be applicable to other biologically active peptides for the development of novel peptide mimetics with high receptor specificity.

## Figures and Tables

**Table 1:** Opioid receptor-binding affinity and biological activities of opioid peptide analogs containing Dmp at position 3/4.

Peptide	Receptor binding affinity, *Ki* ± S. E. (nM)		*δ*/*μ*	*μ*/*δ*	GPI (*μ*)	MVD (*δ*)	MVD/GPI	GPI/MVD
	*μ* ^ a^	*δ* ^b^			IC_50_ (nM)	IC_50_ (nM)		

(1) Leu-enkephalin								
Tyr-Gly-Gly-Phe-Leu (ENK)	2.42 ± 0.93	1.43 ± 0.71	—	1.69	103 ± 30	22.2 ± 4.3	—	4.64
[Dmp^4^]ENK **(10)**	1.25 ± 0.29	17.7 ± 4.2	—	0.07	808 ± 101	624 ± 103	—	1.29
[D-Dmp^4^]ENK **(11)**	2505 ± 169	8924 ± 4098	—	0.28	>10,000	>10,000	—	—
[Dmt^1^]ENK^c^	0.0068 ± 0.003	0.031 ± 0.011	—	0.22	0.55 ± 0.17	0.17 ± 0.02	—	3.24
[Dmt^1^, Dmp^4^]ENK** (12)**	0.030 ± 0.011	0.1 58 ± 0.034	—	0.19	2.00 ± 0.51	1.45 ± 0.26	—	1.38
[Dmt^1^, D-Dmp^4^]ENK **(13)**	5.61 ± 0.59	40.9 ± 11.5	—	0.14	>10,000^d^	>10,000^e^		
(2) Endomorphin-2								
Tyr-Pro-Phe-Phe-NH_2_ (EM2)	0.557 ± 0.306	14,070 ± 3346	25260	—	10.5 ± 1.2	317 ± 65	30.2	—
[Dmp^3^]EM2 **(14)**	0.0304 ± 0.0208	1063 ± 336	34967	—	0.378 ± 0.104	l.39 ± 0.17	3.68	—
[D-Dmp^3^]EM2 **(15)**	2.4 ± 0.56	4169 ± 954	1737	—	30.4 ± 2.8	187 ± 30	6.15	—
[Dmp^4^]EM2** (16)**	13.2 ± 1.9	7624 ± 2571	578	—	196 ± 40	320 ± 55	1.63	—
[D-Dmp^4^]EM2** (17)**	106 ± 20	1765 ± 834	17	—	587 ± 119	2267 ± 603	3.86	—
(3) Demrophin/deltorphin								
Dermorphin (DM)	0.092 ± 0.024	192 ± 51	2087	—	3.74 ± 0.57	34.4 ± 4.8	9.2	—
[Dmp^3^]DM **(18)**	0.00054 ± 0.00021	45.7 ± 11.8	84630	—	1.21 ± 0.23	4.62 ± 0.82	3.8	—
[D-Dmp^3^]DM **(19)**	4.43 ± 1.85	3300 ± 702	745	—	44.4 ± 6.1	358 ± 45	8.1	—
Deltorphin 11 (DT)	314 ± 53	0.0226 ± 0.0077	—	13894	5437 ± 812	0.582 ± 0.029	—	9342
[Dmp^3^]DT **(20)**	1098 ± 111	0.00105 ± 0.00043	—	1045714	6705 ± 992	0.022 ± 0.003	—	304772
[D-Dmp^3^]DT **(21)**	1956 ± 177	111 ± 17	—	18	8214 ± 872	145 ± 15	—	56
(4) Dermorphin-relative short peptide								
Tyr-D-Arg-Phe-*β*Ala-NH_2_ (YRFB)	0.172 ± 0.025	482 ± 121	2802	—	5.31 ± 0.72	116 ± 18	21.8	—
[Dmp^3^]YRFB **(22)**	0.0350 ± 0.0167	544 ± 143	15543	—	1.67 ± 0.24	27.9 ± 5.0	16.7	—
[D-Dmp^3^]YRFB** (23)**	0.0618 ± 0.0109	>2823	>45679	—	19.8 ± 1.9	305 ± 53	15.4	—

^a^Versus [^3^H]DAMGO. ^b^Versus [^3^H]DT. ^c^Data cited from [39]. ^d^Antagonism was shown with pA2 = 6.90 against EM2. ^e^Antagonism was shown with pA2 = 5.57 against DT.

**Table 2:** *K*_e  _ values of opioid receptor antagonists against Dmp-containing YRFB analogs in the GPI and MVD assays.

Peptides		*K*_e_ (nM)	
	GPI	MVD	
	CTAP	CTAP	N,N(Me)_2_Dmt-Tic-OH

[Dmp^3^]YRFB **(22)**	22.7	11.4	>1000
[Dmp^1^]YRFB **(24)**	25.1	13.4	>1000
[Dmp^1,3^]YRFB **(25)**	21.3	10.2	>1000
YRFB	26.8	21.2	>1000
[Dmt^1^]YRFB^b^	85.5	7.89	192
DT	NT^a^	>1000	0.64

^a^Not tested.

**Table 3:** Antinociceptive activities of Dmp-containing YRFB analogs after subcutaneous injection in the formalin test.

Peptides		ED_50_ (95% C. L.)^a^, nmol/kg		
	First phase		Second phase	

[Dmp^3^]YRFB** (22)**	98.6	(26.7–364)	113	(48.6–264 )
[Dmp^1^]YRFB** (24)**	1946	( 1026–3691 )	1529	( 1199–1950)
YRFB	628	(364–1280)	514	(378–700)
Morphine	3811	(2921–4973 )	7319	(4058–13198)

^a^ED_50_ values and 95% confidence limits.

**Table 4:** Receptor-binding affinity of DYN analogs and NOC analogs containing Dmp for opioid receptors and ORLl receptor.

Peptides	IC_50_ ± SEM (nM)				
	ORL1 receptor	Opioid receptor			
	[^3^H]NOC^a^	[^3^H]U-69593 (*κ*)^b^	[^3^H]DAMGO (*μ*)^c^	[^3^H]DT (*δ*)^c^	*κ*/*μ*/*δ*

DYN(I-13)-NH_2_	18.8 ± 3.01	0.162 ± 0.049	2.53 ± 0.38	6.49 ± 1.11	1/15.6/40.1
[Dmp^4^]DYN (1-13)- NH_2_ ** (26)**	188 ± 18.2	0.044 ± 0.035	22.4 ± 10.2	931 ± 723	1/509/21159
[Dmp^1^]DYN(1-13)-NH_2_ ** (27)**	6.60 ± 0.952	0056 ± 0026	16.4 ± 2.35	10.1 ± 6.02	1/293/180
[Dmp^1,4^]DYN (1-13)- NH_2_ ** (28)**	51.5 ± 1.62	5.45 ± 1.65	251 ± 56.3	415 ± 185	1/46/76.1
NOC	0.151 ± 0.058	643 ± 218	1540 ± 601	>10000	—
NOC(1-13)-NH_2_	0.743 ± 0.125	193 ± 54	319 ± 88	>10000	—
[Dmp^4]NOC( 1-13)- NH_2_ ** (29)**	51.6 ± 12.9	299 ± 63	629 ± 433	>10000	—
[Dmp^1^]NOC(1-13)-NH_2_ ** (30)**	0.814 ± 0.090	38.8 ± 16.7	25.0 ± 6.5	292 ± 61	—
[Dmp^l,4^]NOC(1-13)- NH_2_ ** (31 )**	21.3 ± 3.2	100 ± 29	56.8 ± 12.3	3407 ± 990	—

^a^Using cell membrane expressing human ORL1 receptor in Hek-293 cells. ^b^Using guinea pig brain homogenate.

^c^Using rat brain homogenate.

**Table 5:** GPI assay and opioid receptor preference of DYN analogs.

Peptides	IC_50_ ± SEM (nM)	*K*_e_ (nM) value of receptor selective antagonist		
		nor-BNI (*κ*)	CTAP (*μ*)	N,N(Me)_2_Dmt-Tic-OH (*δ*)

DYN(l-13)-NH_2_	3.14 ± 1.13	l.l	99	98
[Dmp^4^]DYN(I-13)-NH_2_ ** (26)**	32.2 ± 9.16	0.63	108	198
[Dmp^1^]DYN(l-13)-NH_2_ ** (27)**	306 ± 68	10	115	>1000
[Dmp^1,4^]DYN(l-13)-NH_2_ ** (28)**	1341 ± 303	809	595	>100

**Table 6:** Opioid receptor-binding affinity and biological activities of opioid peptide analogs containing Dmp at position 1.

Peptide	Receptor binding affinity, *Ki* ± S. E. (nM)				GPI (*μ*)	MVD (*δ*)		
	*μ* ^ a^	*δ* ^b^	*δ*/*μ*	*μ*/*δ*	IC_50_ (nM)	IC_50_ (nM)	MVD/GPI	GPI/MVD

Tyr-Gly-Gly-Phe-Leu (ENK)	2.42 ± 0.93	1.43 ± 0.71	—	1.69	103 ± 30	22.2 ± 4.3	—	4.64
[Dmp^1^]ENK** (32)**	5.94 ± 1.45	1.86 ± 0.61	—	3.19	710 ± 69	66.6 ± 12.7	—	10.66
[Dmt^1^]ENK^c^	0.0068 ± 0.003	0.031 ± 0.011	—	0.22	0.55 ± 0.17	0.17 ± 0.02	—	3.24
[Phe^1^]ENK** (33)**	169 ± 12	73.7 ± 19.5	—	2.29	>10000	>10000	—	—
Deltorphin II (DT)	314 ± 53	0.0226 ± 0.0077	—	13894	5437 ± 812	0.582 ± 0.029	—	9342
[Dmp^1^]DT **(34)**	156 ± 33	0.329 ± 0.077	—	475	4038 ± 1118	0.272 ± 0.054	—	14835
[D-Dmp^l^]DT **(35)**	>2178	1394 ± 495	—	—	>10000	662 ± 147	—	—
[Dmt^1^]DT **(36)**	0.261± 0.060	0.012 ± 0.002	—	21.8	88.4 ± 22.7	0.052 ± 0.007	—	1700
[Phe^1^]DT **(37)**	>2178	2.68 ± 1.22	—	—	>10000	10.4 ± 1.5	—	—
Tyr-Pro-Phe-Phe-NH_2_ (EM2)	0.557 ± 0.306	14,070 ± 3346	25260	—	10.5 ± 1.2	317 ± 65	30.2	—
[Dmp^1^]EM2** (38)**	2.48 ± 1.46	6762 ± 590	2727	—	76.9 ± 20.7	661± 316	8.61	—
[D-Dmp^1^]EM2 **(39) **	40.4 ± 2.6	9714 ± 3820	241	—	1392 ± 221	2329 ± 943	167	—
[Dmt^1^]EM2^d^	0.15 ± 0.04	—	—	—	0.07± 0.02	1.87 ± 0.61	26.7	—
[Phe^1^]EM2 ** (40) **	54.1± 23.4	18,851 ± 10,487	348	—	1073 ± 309	5,199 ± 2,584	4.85	—
Tyr-D-Arg-Phe-*β*Ala-NH_2_ (YRFB)	0.172 ± 0.025	482 ± 121	2802	—	531± 0.72	116 ± 18	21.8	—
[Dmp^1^]YRFB **(24) **	0.0623 ± 0.0140	2572 ± 947	41284	—	9.88 ± 1.04	188 ± 52	19	—
[D-Dmp^1^]YRFB **(41) **	7.62 ± 1.75	>2823	>370	—	320 ± 30	1474 ± 283	4.6	—
[Dmp^1,3^]YRFB ** (25) **	0.0216 ± 0.0062	1688 ± 458	78148	—	2.76 ± 0.56	501 ± 86	18.2	—
[Dmt^1^]YRFB^c^	0.00205 ± 0.00069	1.13 ± 0.13	551	—	0.034 ± 0.065	0.398 ± 0.085	11.7	—
[Phe^1^]YRFB **(42) **	7.17 ± 1.03	>2823	>393	—	633 ± 89	7143 ± 950	11.2	—

^a^Versus [^3^H]DAMGO. ^b^Versus [^3^H]DT. ^c^Data cited from [39]. ^d^Data cited from [29].

**Table 7:** Comparison of stability of Dmp-containing peptides toward enzymatic degradation.

Peptides	Half-life time (min)^a^	
	Aminopeptidase M	Rat brain homogenate

DYN(I-13)-NH_2_	15.5	435
[Dmp^4^]DYN(l-13)-NH_2_ ** (26)**	NT^b^	315
[Dmp^1^]DYN(1-13)-NH_2_ ** (27)**	>30	577
[Dmp^1,4^]DYN(1-13)-NH_2_ ** (28)**	NT^b^	770
NOC(1-13)-NH_2_	12	41.5
[Dmp^4^]NOC(1-13)-NH_2_ ** (29)**	NT^b^	33.6
[Dmp^1^]NOC(1-13)-NH_2_ ** (30)**	28	60.3
[Dmp^1,4^]NOC(l-13)-NH_2_ ** (31)**	NT^b^	27.1
Met-enkephalin	<5	8.5

^a^Determined by HPLC. ^b^Not tested.

## Abbreviations

Dmp: 2′,6′-Dimethylphenylalanine
Dmt: 2′,6′-Dimethyltyrosine
DAMGO: [D-Ala^2^, MePhe^4^, Gly-ol^5^]enkephalin
ENK: Leu-enkephalin, Tyr-Gly-Gly-Phe-Leu
DM: Dermorphin Tyr-D-Ala-Phe-Gly-Tyr-Pro-Ser-NH_2_
DT: [D-Ala^2^]deltorphin II, Tyr-D-Ala-Phe-Glu-Val-Val-Gly-NH_2_
YRFB: Tyr-D-Arg-Phe-*β*Ala-NH_2_
EM2: Endomorphin 2, Tyr-Pro-Phe-Phe-NH_2_
CTAP: D-Phe-c[Cys-Tyr-D-Trp-Arg-Thr-Pen]-Thr-NH_2_
NOC: Nociceptin/orphanin FQ
DYN: Dynorphin A
ORL1: Opioid receptor-like 1
AP-M: Aminopeptidase M
GPI: Guinea pig ileum
MVD: Mouse vas deferens.
